# Pathways to catastrophic health expenditure for acute coronary syndrome in Kerala: ‘Good health at low cost’?

**DOI:** 10.1186/1471-2458-12-306

**Published:** 2012-04-26

**Authors:** Meena Daivadanam

**Affiliations:** 1 Achutha Menon Centre for Health Science Studies (AMCHSS), Sree Chitra Tirunal Institute for Medical Sciences and Technology (SCTIMST), Thiruvananthapuram, 695011, India; 2 Dept. of Public Health Sciences, Division of Global Health (IHCAR), Karolinska Institutet, Nobels väg 9, Stockholm, SE 171 77, Sweden

**Keywords:** Catastrophic health expenditure, Distress financing, Acute coronary syndrome, Out-of-pocket expenditure, Non-communicable diseases, Universal health coverage

## Abstract

**Background:**

Universal health coverage through the removal of financial and other barriers to access, particularly for people who are poor, is a global priority. This viewpoint describes the many pathways to catastrophic health expenditure (CHE) for patients with Acute Coronary Syndrome (ACS) based on two case studies and the thematic analysis of field notes regarding 210 patients and their households from a study based in Kerala, India.

**Discussion:**

There is evidence of the severe financial impact of non-communicable diseases (NCDs), which is in contradiction to the widely acclaimed Kerala model: Good health at low cost. However, it is important to look beyond the out-of-pocket expenditure (OOPE) and CHE to the possible pathways and identify the triggers that make families vulnerable to CHE. The identified pathways include a primary and secondary loop. The primary pathway describes the direct path by which families experience CHE. These include: 1) factors related to the pre-event period that increase the likelihood of experiencing CHE, such as being from the lower socio-economic strata (SES), past financial losses or loans that leave families with no financial shock absorber at the time of illness; 2) factors related to the acute event, diagnosis, treatment and hospitalization and expenditures incurred for the same and; 3) factors related to the post-event period such as loss of gainful employment and means of financing both the acute period and the long-term management particularly through distress financing. The secondary pathway arises from the primary and includes: 1) the impact of distress financing and; 2) the long- and short- term consequences of CHE. These factors ultimately result in a vicious cycle of debt and poverty through non-compliance and repeat acute events.

**Summary:**

This paper outlines the direct and indirect pathways by which patients with ACS and their families are trapped in a vicious cycle of debt and poverty. It also contradicts the prevailing impression that only low-income families are susceptible to CHE, distress financing and their aftermaths and underscores the need for a deeper understanding at the micro-level, if Kerala and India as a whole are to undertake the difficult exercise of achieving universal health coverage to successfully tackle its growing NCD burden.

## Background

‘Universal health coverage through removal of financial and other barriers to access, particularly for people who are poor, is a priority but political commitment will be needed’ [1]. This statement from a background paper for the 2010 Global Symposium on Health System Research and reiterated in the Lancet article ‘Priority actions for Non-communicable disease crisis’ [2] embodies both the critical gap as well as the glaring reality associated with financial protection in most low- and middle- income countries (LMICs) including India. While this is true for any illness, chronic non-communicable diseases (NCDs) like coronary artery disease (CAD), stroke and diabetes, with their expensive treatment options (particularly for acute events), life-long medication and reduced earning potential, extract a higher toll from patients and their families [3-5]. In India, NCDs accounted for 53% of all deaths and 44% of Disability-Adjusted Life-Years in 2005 with the lower socio-economic strata (SES) at higher risk [6]. Moreover, premature NCD mortality (< 60 years), which affects the most productive age groups, accounted for a major portion of total NCD deaths (males – 38%, females – 32%) [7].

The macro-economic impact of NCDs have been studied extensively [8,9] but studies detailing their effect at the micro-level largely rely on national household surveys or are confined to a few diseases in relatively small settings [3,10]. Furthermore, studies looking at the impact of catastrophic health expenditure (CHE) at this level are fewer still [11]. This micro-view is important in order to understand both, where the different strategies to address CHE should be targeted and how it will affect the concerned individuals and their families. Hence, the objective of this paper is to describe the many pathways to CHE for patients with Acute Coronary Syndrome (ACS) – the major cause of deaths in CAD. While doing so, I would like to argue that financial protection, which includes financial access, is one of the most important deciding factors in the treatment and management of acute events related to NCDs.

## Discussion

### 1. Out-of-pocket and catastrophic health expenditure for ACS

**Figure 1:**
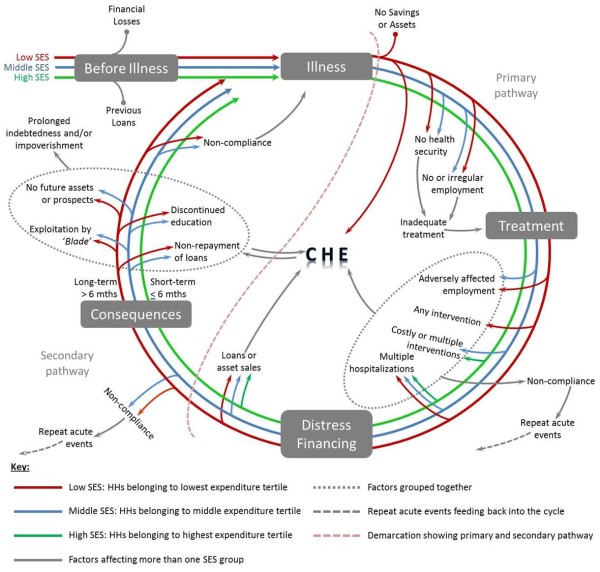
**Pathways to Catastrophic Health Expenditure.** This figure describes the primary and secondary pathways by which low-, middle-, and high-income households experience CHE and get trapped in a vicious cycle of illness, debt and poverty. Households enter this cycle through their first acute coronary syndrome (ACS) event. The cycle is divided into four segments: 1) illness - treatment: factors that prevent households from receiving adequate treatment; 2) treatment - distress financing: aspects related to treatment financing particularly with respect to hospitalization and interventions as well as the adverse effect of the illness on employment, all of which tend to increase non-compliance and lead to repeat acute events; 3) distress financing - consequences: means of distress financing that increase the intensity and duration of CHE; 4) consequences – illness: short- and long-term consequences of CHE experienced by households in their journey through the cycle. The events grouped together in this segment aggravate CHE as the cycle repeats itself, at the same time, households experiencing CHE are at higher risk of undergoing these consequences leading to prolonged indebtedness and (or) impoverishment (depicted by ⇆).

This is a viewpoint arising mainly from analysis of qualitative data related to a study on out-of-pocket expenditure (OOPE) from 210 cases of ACS in Kerala State. ACS-related direct and indirect expenditure data were collected for a nine-month period: three months pre-event and six months post-event and OOPE was estimated for the same period [12]. In addition to the quantitative data, detailed field notes were maintained for each of the study participants regarding: 1) family circumstances prior to the illness; 2) coping strategies used by the family to handle the increased financial burden and; 3) the problems encountered while accessing health care, arranging finances and during the follow-up period. Two case studies were also recorded and transcribed verbatim after obtaining appropriate informed consent. Thematic analysis was done on both the field notes and the case studies [13]. Emerging descriptive themes were coded and analyzed within the broad framework of pre-event, acute event and post-event periods. Themes and their categories were then interlinked to identify the various pathways that lead to CHE as shown in Figure 1.

CHE for the purpose of this study was defined as ACS-related expenditures exceeding 40% of a household’s annual non-food expenditure [14]. Median ACS-related expenditure was 116,795 INR ~ 2,570 USD (Range: 275 – 19,550 USD) and CHE was experienced by as many as 84% of study participants including all socio-economic strata. [12] Study participants belonging to the low SES were 15 times more likely to have experienced CHE compared to their counterparts (odds ratio (OR): 14.51, 95% Confidence Interval (CI): 1.69-124.41). Those whose jobs were adversely affected were seven times more likely (OR: 7.21, CI: 1.54-33.80), those who had no health security were six times more likely (OR: 6.00, CI: 2.02-17.81) and those who underwent any intervention were three times more likely to have experienced CHE (OR: 3.24, CI: 1.03-10.16). [12] ACS imposed large financial burdens on families, with OOPE up to 9 times their total household expenditures, depending on their socio-economic status and type of treatment availed. In fact the magnitude of the OOPE is such that only a small proportion managed to limit their ACS-related expenditure within their overall household expenditure. This included those from the high SES, those who had some form of health security coverage for their illness and those who did not have to resort to distress financing to meet their treatment expenses.

The high OOPE and CHE are no doubt critical findings that need to be tackled seriously. However, in order to do that, it is important to identify the triggers that make families vulnerable to CHE and hasten their transition from ‘high’ to ‘catastrophic’. Understanding these pathways is essential if our policies and strategies are to be targeted effectively.

### 2. Pathways to CHE

The pathways to CHE arise from varied circumstances and time points and can be described in terms of a primary and secondary pathway (Figure 1).

#### Primary pathway

The primary pathway describes only those sets of circumstances or factors that directly lead to households experiencing CHE. It can be predominantly explained in terms of the following circumstances: financial status of the household prior to the illness; employment status of the patient and household and effect of illness on the employment; presence or absence of health security; treatment requirement in terms of hospitalizations and intervention and; the means of treatment financing and in particular, the need to resort to distress financing. These have been well documented in India and other settings as well [11,15-18].

Many families are already vulnerable to CHE when they become ill: if they are from the lower SES, have no assets or have incurred financial losses or taken out previous loans to meet costs for education or marriage of children or treatment of other family members. This is aggravated by the acute event and the strain of having to raise a substantial amount for treatment in a relatively short time.

Only 29% of the study households had some form of health security coverage (partial or complete) for their ACS episode(s) while 70% had resorted to loans or asset sales to finance their treatment of which 50% were first-time applicants [12]. Acute events like ACS are sudden and often traumatic, leaving families helpless and desperate. Individuals and families are caught unaware as treatment options are expensive and their savings or assets are usually insufficient. In the rush to mobilize immediate funds, they opt for quick solutions, i.e., distress financing, often from illegitimate sources. Legitimate financial institutions require time, endless paperwork and the possession of demonstrable assets. On the other hand, the extensive subculture of local moneylenders who are known in Kerala slang as *‘Blade’*, because of their rather cut-throat tactics, require no processing time but charge high daily interest rates. This type of loan does not even provide temporary respite as interest is accrued daily and there is constant pressure to repay the interest amount to keep the loan viable. With regards to employment, ACS rendered a few individuals incapable of continuing their jobs; while many employers forced their ill employees to retire, fearing loss of productivity; which tends to further push families into a vicious cycle of ill health, distress financing & poverty.

#### Secondary pathway

The secondary pathway is a by-product of the primary pathway and includes the impact of distress financing and the short- and long-term consequences of CHE.

In a predominantly collectivist culture such as that found in India including Kerala, the burden of ill-health tends to be shared among all household members albeit unequally. While all family members are forced to bear the brunt of such a misfortune, the more vulnerable members in the equation, particularly women and children, tend to take on the burden disproportionately [19]. Many children discontinued their education and started working to add to the family income. Many spouses also started working for the first time in their lives. Families moved out of expensive rental accommodation to cheaper or ‘free’ accommodation with obliging relatives and children were transferred to cheaper schools which were perceived to offer only sub-standard education.

Households used multiple cost-cutting strategies like those mentioned above to adjust their expenses even in the short six-month post-event period. Non-compliance was another form of ‘adjustment’ which involved choosing a cheaper treatment alternative or forgoing long-term medications that is the mainstay of any chronic disease management. Thus, families became further susceptible to the threat of future acute events owing to the lack of treatment or follow-up medication, ultimately leading to a never-ending vicious cycle. (Figure 1).

In the case of distress financing via local moneylenders, exploitation was frequent, as property deeds or other assets that guarantee these transactions were seized when families were unable to repay. This leads to life-time indebtedness in many cases which may even be passed on to the next generation, particularly among the low SES. They deplete not just their emergency funds, but also their future prospects when children have had to drop out of school. Many of them also faced the stigma of being labeled a ‘charity case’ by exasperated relatives.

In addition, there are other factors which exacerbate this vicious cycle (Figure 1). The emotional trauma of having to cope with the illness, the resulting financial and mental exhaustion, the distress of watching their families struggle to survive and make ends meet in the aftermath of the illness and its treatment, the social isolation that results from a fear among relatives that such an individual may become a burden to them and feeling of utter helplessness and hopelessness when they foresee no end to their struggles.

These tangible and intangible pathways to CHE, including powerful emotions that cannot be quantified, are best illustrated by means of the quite contrasting stories of two men who were diagnosed to have ACS, Mr. Mani and Mr. Kumar (names changed to ensure confidentiality).

### 3. CHE: two contrasting perspectives

#### *i.* Am I a charity case?

"“*I am not sure how long I can take my medicines. I have a credit account with the local pharmacy. They also help me out with samples from medical representatives. I cannot be a charity case forever, can I?”* (Quote by Mr. Mani, 50 yrs. ACS patient*)*"

Mr. Mani (50 yrs.) was diagnosed with ACS at a public hospital and initially refused to undergo an angioplasty due to lack of financial resources. However, a second episode within days forced him to undergo the intervention on an emergency basis. His treatment was financed solely through *‘Blade’* money which he could not repay (79,567 INR ~ 1,751 USD). His illness left him unable to continue his previous work; carrying loads which earned him an average of INR 775 ~ 17 USD daily. He could barely walk without chest pain, but still went to the ‘agarbatti’ (incense stick) rolling factory where he did supervisory work for 30 INR ~ 0.7 USD a day.

"“*How can I sit at home? Even my daughter, who is still studying, now gives tuition before she goes for her classes and my wife has also started working. So, I have to at least do my share*.” “*Right now, I am staying with one of my sisters, so that I don’t have to pay rent, water or electricity charges. My other sister has cut all ties with me. She fears that I will become a burden on her and her family. Can I really blame her*?” *(*Quote by Mr. Mani, 50 yrs. ACS patient*)*"

#### *ii.* Could I have managed otherwise?

"“*I don’t have to pay even a rupee for my medicines. When the local pharmacist told me that my monthly medicines cost around INR 3,000, I got a shock. I don’t know how I would have managed if I had had to pay for it myself*.” (Quote by Mr. Kumar, 66 yrs. ACS patient)"

Mr. Kumar (66 yrs.), a retired army man, had been estranged from his children for a few years. A widower, he found refuge in an old people’s home in the city following his wife’s suicide two years ago. His monthly pension of INR 2,000 ~ 44 USD is sufficient to manage his day-to-day life including his room rent and food. When he suddenly fell ill, he had to undergo an angiogram and angioplasty, all within the space of five days for which he spent 185,580 INR ~ 4,085 USD. Being a retired government servant from the armed forces, a category of government employees who enjoy the best social security benefits in the country, his treatment was financed directly by the government office concerned. This also covered his regular follow-up visits including his monthly medications.

According to Mr. Kumar, managing his daily life simultaneously with his illness would have been impossible on his meager pension alone. Predictably, he has to contend with bureaucratic hurdles which he describes as a ‘small’ price for the privilege of being stress-free.

No doubt, these two stories represent the extreme ends of the CHE spectrum. With the highest CAD prevalence in India (rural - 7.5%, urban – 12%) [20], Kerala has many such stories that will provide the in-between shades of this spectrum. But they do not explain the ‘why’, which may be better answered through some background facts related to the Kerala health system.

### 4. CHE and the Kerala health system

The high OOPE and CHE have been reported elsewhere with respect to CVD-related hospitalizations (including ACS) where India (Kerala) had the highest 15-month OOPE and more than 80% of the low- and middle- income groups and more than 60% of the high income group experienced CHE [4]. These figures are also reflected in the health financing data which shows that Kerala state has the highest private expenditure (90.3%) compared to any other state in India [21]. Since the relationship between OOPE, NCDs and CHE has been long established [5], these findings are to be expected considering the high prevalence of NCDs and their risk factors in the state [20,22,23].

This evidence of severe financial impact is perhaps surprising for some as the Kerala health system was once advocated as the ideal -‘Good Health at Low Cost’ model [24]. It has however come under considerable pressure as a result of the combination of low public health spending and the steep rise in healthcare costs, especially for NCDs. The lax regulations in establishing private healthcare institutions, the dramatic rise in disposable income among the population, their changing expectations and consumerist attitudes have all contributed to this deplorable state of affairs [25]. Moreover, as is the case with the rest of India, primary healthcare is free only in principle. The poor can theoretically opt for cheaper or ‘free’ in-patient care through price discrimination [26]. However, facilities for the treatment of NCDs and their acute events are few in the public sector and the available facilities are not completely free even for the poor. Moreover, critical medicines are often unavailable and under-the-table, informal payment practices are rampant [27,28]. Kerala’s advanced stage of epidemiological transition [22] further compounds the problem with NCDs like CAD becoming more prevalent, particularly among the poor.

This study highlights some of the possible background factors pertaining to the Kerala health system that may have contributed to its failure to protect its citizens from the financial consequences of seeking treatment. These factors, namely inadequate budgetary provision for health [29], lack of preparedness of the public health system to face the current and upcoming NCD burden [30,31] and lack of an effective mechanism to counter CHE, are interlinked and have been identified both in India and other settings [32]. The findings of this study presented here as well as elsewhere [12] demonstrate that while it is important to provide essential drugs and the best treatment options, it is far more important to ensure that these are affordable and accessible to all. While considering the treatment acquisition process, financial access and protection are akin to the basic physiological needs described in Maslow’s hierarchy of needs [33]. Once this need is met, other factors will surface, but at present the basic need is paramount.

First, the budgetary provision for health in Kerala state is grossly inadequate [34]. Consequently, the public services are under-funded. With deaths from NCDs projected to increase substantially in the coming years, the scale of CHE is likely to spin out of control. While individuals and households are largely burdened with this cost of care, the cost to the state machinery will be staggering if nothing is done, as it will also include the loss of productive man-hours. Health is the responsibility of the individual state governments in India. Hence, it becomes imperative that states fall in line with the commitment of the Indian government to increase public spending on health from less than 1% to 3% of Gross Domestic Product (GDP) [21].

Second, the public health system in Kerala, one of the most advanced in India, is not geared towards the management of NCDs and is unable to mitigate the consequences of seeking treatment. The government health services are less prepared to manage the complications of NCDs than the for-profit sector. The for-profit health services have fully capitalized on the government’s inability to recognize the growing NCD burden in the state and filled that gap [35], extracting a disproportionately high price from all but the very rich. They are over-endowed with up-to-date technology and have world class facilities thus resulting in substantial overheads and ordinarily such centers would be patronized only by the upper economic classes, i.e., those who can afford it. However, that is not the case in Kerala as even the poor are forced to turn to them in the absence of accessible public health services [26,36]. Unfortunately, this has come with a price, both literally and figuratively speaking. In other words, Kerala has started to sacrifice the welfare gains of the past and the market is gradually taking over the health sector [25]. This needs to be reversed. The Kerala public health system cannot just sit back and wait until it is completely sidelined by the for-profit sector [26,35]. It is time for an overhaul, and a strengthening of the public health system in Kerala, not unlike the one that the Lancet authors of the call to action “Towards universal health coverage by 2020” have in mind for the whole of India [37]. The public health system should be the primary provider of promotive, preventive, curative and rehabilitative health services, to improve quality and reduce the OOPE on health care. Where necessary, the for-profit sector should be regulated and integrated within the health care system [35].

Third, taking into consideration the chronic nature of NCDs and the various costs incurred by the families, a viable financing mechanism is warranted to avert CHE. A part of the solution, as stated above, lies in increasing the government spending on health. Kerala should give top priority to financial protection for its citizens on the health sector agenda. While the new national health insurance initiative, called the RSBY-CHIS (Rashtriya Swasthya Bima Yojna – Comprehensive Health Insurance Scheme) [38] which is currently being implemented in the state through private insurance providers, is a step in the right direction, it is unfortunately not grounded in reality. Neither Kerala’s disease profile with its distinct leaning towards the NCD end of the spectrum [20,22,23] nor the historical lessons regarding serious equity compromises related to private health insurance have been given due consideration [39]. The Kerala healthcare stakeholders should be serious in their efforts to curb OOPE and the increased health sector funding will have to be paid through pre-payment schemes or higher taxes.

Admittedly, Kerala is an outlier in India. It is one of the most educationally forward states in India (including female literacy) and its major health indicators are comparable to the developed Western world. [24] While Kerala has been a role model for India as well as the whole world through many of its achievements, in the NCD context, it is a harbinger of disasters to come unless trends and behaviour can be broken or changed. So, while Kerala is currently not representative of the rest of India, it has important lessons to learn and to impart to the country as a whole, as well as to other LMICs through its struggle to place the growing NCD epidemic and the need for universal coverage within an appropriate framework.

Health is the basic right of an individual. ‘Health for All’ is of course what we work towards and hope for, but ‘Healthcare for All’ may be a more attainable goal. As seen in this study, direct payments at the point of entry will create serious problems for all but the most wealthy. Considering the evidence available from around the globe, universal health coverage is therefore the way to go [2,40]. It is feasible in various settings, with relatively limited resources. Countries as diverse as the Czech Republic, Slovakia, Rwanda and Thailand [14,22,41,42] are showing the way. We need to study how and why it worked there and what we need to do to make it work for us, taking into account our basic realities as well as our fundamental differences. It is imperative that Kerala and India as a whole undertake the difficult challenge of universal health coverage if it is to successfully tackle its growing NCD burden.

## Summary

This paper looks beyond the evidence of the severe financial impact of ACS at the micro-level and attempts to map out the various pathways by which households experience CHE. The primary pathway describes the direct path by which families experience CHE. These include: 1) factors related to the pre-event period that increase the likelihood of experiencing CHE, such as being from the lower SES, past financial losses or loans that leave families with no financial shock absorber at the time of illness; 2) factors related to the acute event, diagnosis, treatment and hospitalization and expenditures incurred for the same and; 3) factors related to the post-event period such as loss of gainful employment and means of financing both the acute period and the long-term management particularly through distress financing. The secondary pathway arises from the primary and includes: 1) the impact of distress financing and; 2) the long- and short- term consequences of CHE. These factors ultimately loop back into a vicious cycle of debt and poverty through non-compliance and repeat acute events. It further contradicts the prevailing notion that only low-income families are susceptible to CHE, distress financing and their aftermaths, particularly for acute events related to NCDs and emphasises the need for a deeper understanding at the micro-level if we are to arrive at viable solutions that will benefit the vast majority.

## Abbreviations

CHE, Catastrophic health expenditure; ACS, Acute coronary syndrome; NCDs, Non-communicable diseases; LMICs, Low- and middle- income countries; CAD, Coronary artery disease; OOPE, Out-of-pocket expenditure; SES, Socio-economic status; RSBY-CHIS, Rashtriya Swasthya Bima Yojna – Comprehensive Health Insurance Scheme; GDP, Gross Domestic Product; OR, Odds ratio; CI, Confidence interval; INR, Indian Rupees; USD, US Dollars.

## Competing interests

The author declares that she has no competing interests.

## Authors’ contributions

MD is the sole author.

## Pre-publication history

The pre-publication history for this paper can be accessed here:

http://www.biomedcentral.com/1471-2458/12/306/prepub
